# Mining biosynthetic gene clusters in *Paenibacillus* genomes to discover novel antibiotics

**DOI:** 10.1186/s12866-024-03375-5

**Published:** 2024-06-27

**Authors:** Man Su Kim, Da-Eun Jeong, Jun-Pil Jang, Jae-Hyuk Jang, Soo-Keun Choi

**Affiliations:** 1 Infectious Disease Research Center, Korea Research Institute of Bioscience and Biotechnology (KRIBB), Daejeon, Republic of Korea; 2 Department of Biosystems and Bioengineering, KRIBB School of Biotechnology, University of Science and Technology (UST), Daejeon, Republic of Korea; 3 Chemical Biology Research Center, Korea Research Institute of Bioscience and Biotechnology, Cheongju, Republic of Korea; 4 Department of Applied Biological Engineering, KRIBB School of Biotechnology, University of Science and Technology (UST), Daejeon, Republic of Korea

**Keywords:** Biosynthetic gene cluster, *Paenibacillus*, Genome mining, Antibiotics, Cytosine base editor, Antibiotic dereplication

## Abstract

**Background:**

Bacterial antimicrobial resistance poses a severe threat to humanity, necessitating the urgent development of new antibiotics. Recent advances in genome sequencing offer new avenues for antibiotic discovery. *Paenibacillus* genomes encompass a considerable array of antibiotic biosynthetic gene clusters (BGCs), rendering these species as good candidates for genome-driven novel antibiotic exploration. Nevertheless, BGCs within *Paenibacillus* genomes have not been extensively studied.

**Results:**

We conducted an analysis of 554 *Paenibacillus* genome sequences, sourced from the National Center for Biotechnology Information database, with a focused investigation involving 89 of these genomes via antiSMASH. Our analysis unearthed a total of 848 BGCs, of which 716 (84.4%) were classified as unknown. From the initial pool of 554 *Paenibacillus* strains, we selected 26 available in culture collections for an in-depth evaluation. Genomic scrutiny of these selected strains unveiled 255 BGCs, encoding non-ribosomal peptide synthetases, polyketide synthases, and bacteriocins, with 221 (86.7%) classified as unknown. Among these strains, 20 exhibited antimicrobial activity against the gram-positive bacterium *Micrococcus luteus*, yet only six strains displayed activity against the gram-negative bacterium *Escherichia coli*. We proceeded to focus on *Paenibacillus brasilensis*, which featured five new BGCs for further investigation. To facilitate detailed characterization, we constructed a mutant in which a single BGC encoding a novel antibiotic was activated while simultaneously inactivating multiple BGCs using a cytosine base editor (CBE). The novel antibiotic was found to be localized to the cell wall and demonstrated activity against both gram-positive bacteria and fungi. The chemical structure of the new antibiotic was elucidated on the basis of ESIMS, 1D and 2D NMR spectroscopic data. The novel compound, with a molecular weight of 926, was named bracidin.

**Conclusions:**

This study outcome highlights the potential of *Paenibacillus* species as valuable sources for novel antibiotics. In addition, CBE-mediated dereplication of antibiotics proved to be a rapid and efficient method for characterizing novel antibiotics from *Paenibacillus* species, suggesting that it will greatly accelerate the genome-based development of new antibiotics.

**Supplementary Information:**

The online version contains supplementary material available at 10.1186/s12866-024-03375-5.

## Background

A recent survey, encompassing 471 million individual records/isolates from 204 countries and territories, revealed that bacterial antimicrobial resistance (AMR) caused 4.95 million deaths [1]. AMR poses a severe threat to humanity, necessitating the urgent development of new antibiotics. Traditional methods for antibiotic discovery involving activity screening of soil-derived microorganisms have been abandoned owing to the re-discovery of known compounds [2]. Alternative methods, such as developing synthetic antibiotics through high-throughput screening and rational drug design, have limitations, mainly related to inadequate penetration of these antimicrobial agents through the bacterial cell wall and their narrow antimicrobial spectrum [2]. Recently, machine learning methods have been introduced to expedite the exploration of chemical libraries for discovering novel antibiotics [3]. Screening for new antibiotics from previously inaccessible or underexplored microbial sources has been successful [4, 5]. However, this approach remains challenging due to the presence of a large number of previously discovered compounds [6]. Dereplication, the elimination of known antibiotics in microbial extracts, is a laborious and time-consuming process. Advanced techniques such as mass spectrometry and nuclear magnetic resonance-based metabolomics have been introduced to assist in dereplication [7]. Nonetheless, they often require pure fractions, rendering them unsuitable for initial screening. Thus, a new platform is needed for antibiotic discovery.

Recent advances in genome sequencing have unveiled a wealth of untapped biosynthetic gene clusters (BGCs) in microbial genomes, offering new avenues for antibiotic discovery [8, 9]. Actinobacteria, renowned for their contributions to traditional antibiotics, have been the focus of large-scale genome mining efforts due to their diverse BGCs [9, 10]. Additionally, bacterial species in the order Bacillales have garnered attention as a resource for novel antibiotics [11–13]. They produce antibiotics structurally distinct from Actinobacteria. Among the Bacillales members, antibiotic studies have focused on the family Bacillaceae, whereas strains belonging to the family Paenibacillaceae have been insufficiently explored. Our analysis of *Paenibacillus* genomes from the National Center for Biotechnology Information (NCBI) database revealed that 84.4% of the BGCs were uncharacterized. The result underscores the potential of *Paenibacillus* species as sources for discovering new antibiotics. Moreover, our research demonstrates that new antibiotics can be efficiently and rapidly characterized via cytosine base editor (CBE)-mediated genetic dereplication in a selected *Paenibacillus* strain.

## Methods

### Strains and culture conditions

The strains utilized in this study are detailed in Supplementary Table S1. For general cloning, *Escherichia coli* strain MC1061 [14] was employed. *Paenibacillus* strains were sourced from the Korean Agricultural Culture Collection (KACC) and the Korean Collection for Type Cultures (KCTC). *Escherichia coli* and *Bacillus subtilis* strains were cultured in Luria-Bertani medium (LB; Difco, Detroit, MI, USA) at 37 °C. *Paenibacillus* strains were cultivated in tryptic soy broth (TSB; Difco) or tryptic soy agar (TSA) at 30 °C. When necessary, the medium was supplemented with chloramphenicol (7.5 µg/mL for *Paenibacillus brasilensis*) or ampicillin (100 µg/mL). *P. brasilensis* transformation was conducted as previously described [15]. Indicator strains from the American Type Culture Collection (ATCC), KCTC, or KACC for antimicrobial activity assays were cultured as follows: *Bacillus cereus* was grown in LB broth or agar at 30 °C; *E. coli* and *Acinetobacter baumannii* were grown in LB broth or agar at 37 °C; *Micrococcus luteus* was grown in TSB or TSA at 30 °C; *Pseudomonas aeruginosa* was grown in TSB or TSA at 37 °C; *Pythium ultimum*, *Fusarium graminearum*, and *Rhizoctonia solani* were grown in potato dextrose agar (PDA; Difco) at 25 °C.

### *Paenibacillus* genome sequence collection and BGC analysis

A total of 554 *Paenibacillus* genome sequences were acquired from the NCBI Genome Sequence Database. BGCs for secondary metabolites were predicted using antiSMASH v6.1.1 [16]. Distinguishing existing and new BGCs was based on BGC type, predicted domain, and knownClusterBlast confirmed using AntiSMASH.

### Construction of strain *P*. *brasilensis* RB5

The plasmids and primers used in this study are listed in Supplementary Tables S2 and S3, respectively. Plasmid pMisCBE4-RB5 was constructed to inactivate four BGCs in *P. brasilensis*, except BGC5. Primer sets BB-vec-sgF/Bsa-sgR1, Bsa-sgF1/Bsa-sgR2, Bsa-sgF2/Bsa-sgR3, and Bsa-sgF3/SCBB-vec-sgR were used to amplify the BGC1a-, BGC1b-, BGC3-, and BGC11-targeting single-guide RNA (sgRNA) cassettes, respectively. The amplified sgRNA cassettes and backbone plasmid pMGoldi-sCBE4 [15] were assembled using a modified Golden Gate assembly protocol [17] to construct pMisCBE4-RB5. *P. brasilensis* was transformed with pMisCBE4-RB5 using the previously described conjugation method, the modified integrative and conjugative element (MICE) [15]. Randomly selected transformants were analyzed using DNA sequencing to confirm the mutations. Curing of plasmid pMisCBE4-RB5 was performed using a previously described method [17] to generate *P. brasilensis* RB5. The RB5 strain was confirmed to harbor the relevant mutations via DNA sequencing.

### Construction of *P*. *brasilensis* RB5d5

The remaining BGC5 was deleted from the RB5 strain to construct the RB5d5 strain using a *Bacillus* integrative plasmid combining a synthetic gene circuit (BIPS) system reported previously [18]. To construct a plasmid for deleting BGC5, two homologous arm fragments corresponding to the upstream and downstream regions of the target gene were amplified from the chromosome of *P. brasilensis* using the primer sets 1G-BRB5-FF/1G-BRB5-FR and 3GX-BRB5-BF/3GX-BRB5-BR, respectively. The chloramphenicol resistance gene (*cat*) under the control of the P_*spac*_ promoter (P_*spac*_-*cat* fragment) was amplified from plasmid pA-xylR2 [19] using the primer set Pspac-F/cat-R. The homologous arm and P_*spac*_-*cat* fragments were cloned into pSGC4iN [18] using a modified Golden Gate assembly protocol to construct pSGC4iN-RB5d. After introducing pSGC4iN-RB5d into the conjugation donor MICEaRep [18], the plasmid was transformed into the recipient *P. brasilensis* RB5 using the MICE method to construct *P. brasilensis* RB5d5. The BGC5 mutation in RB5d5 was confirmed via sequencing.

### Antimicrobial activity assay

The indicator strains were cultured for 16 h in the appropriate medium at 30 °C for *B. cereus* and *M. luteus* and at 37 °C for the other strains. Antimicrobial assay plates were prepared using LB or TSA agar containing a 1% culture of each indicator strain. *Paenibacillus* strains were grown in 2 mL of TSB at 30 °C for 16 h. After adjusting the cultures to an optical density at 600 nm (OD_600_) of 2 in TSB medium, 5 µL of the cultures was spotted on each bioassay plate followed by incubation for 24 h (48 h for *M. luteus*) at 30 °C for *B. cereus* and *M. luteus* and 37 °C for the other strains. Subsequently, the diameters of the inhibition zones (mm) were measured. For methanol extraction, *Paenibacillus* strains were cultured in TSB at 30 °C for 24 h. Methanol extraction and antimicrobial activity assays of the extracts were performed using a previously reported method [20].

### Antifungal activity assay

All fungi were grown on PDA plates at 30 °C for 3 days. Subsequently, 5 mm plugs of each fungus were placed in the center of a new PDA plate. *Paenibacillus* strains were cultured in 2 mL of TSB at 30 °C for 16 h. After adjusting the cultures to OD_600_ of 2 in TSB medium, 5 µL of the cultures was spotted 2 cm away from the plug. The plates were incubated at 30 °C for 3 days.

### Liquid chromatography–mass spectrometry (LC/MS) analysis

An amount of 1 µl of the methanol extracts was injected into an Ultra Performance Liquid Chromatography Ethylene-Bridged Hybrid (UPLC BEH) C18 Column (1.7 μm particle size, 100 mm length x 2.1 μm inner diameter) (Waters Corporation, Drinagh, Ireland) and separated using a Shimadzu LCMS-8050 triple-quadrupole mass spectrometer (Shimadzu Corporation, Kyoto, Japan). Solvent A was 0.1% formic acid in water. Solvent B was 0.1% formic acid in acetonitrile. The following gradient conditions were used for the chromatography: 0–0.01 min, 5% B; 0.01–2.0 min, linear gradient, 5% B; 2.0–15.0 min, 80% B; 15.5–20.0 min, 100% B; 20.0–20.5 min, 5% B; and re-equilibration at 5% B for 3.5 min. The flow rate was 0.3 mL/min.

### Purification and structure determination of the novel compound

*P. brasilensis* RB5 was grown in a 250 mL Erlenmeyer flask with 50 mL of tryptic soy broth (TSB; Difco) seed culture medium for 24 h at 30 °C on a rotary shaker set at 180 rpm. For larger-scale cultivation, 1% of the seed culture was transferred into 40 baffled 1,000 mL Erlenmeyer flasks containing 250 mL of TSB broth. These were cultured for 48 h at 30 °C on a rotary shaker at 180 rpm. The resulting residue was partitioned with ethyl acetate three times and then evaporated to remove the solvent. The crude extract was fractionated using reversed-phase C_18_ vacuum column chromatography with a stepwise solvent system of methanol and water (20:80 to 100:0 v/v, each in 1-liter volumes). The 80% fraction (120 mg) was further purified by reversed-phase HPLC (Cosmosil semipreparative C_18_, 30% acetonitrile, 3 mL/min, UV detection at 210, 265 nm) to obtain the desired compound. NMR spectra were recorded on Bruker AVANCE HD 900 NMR spectrometers at the Korea Basic Science Institute (KBSI) in Ochang, South Korea. Chemical shifts were referenced to the residual solvent signal (DMSO-*d*_6_; *δ*_H_ 2.50, *δ*_C_ 39.51).

## Results

### Mining of BGCs in *Paenibacillus* genomes 

**Fig. 1:**
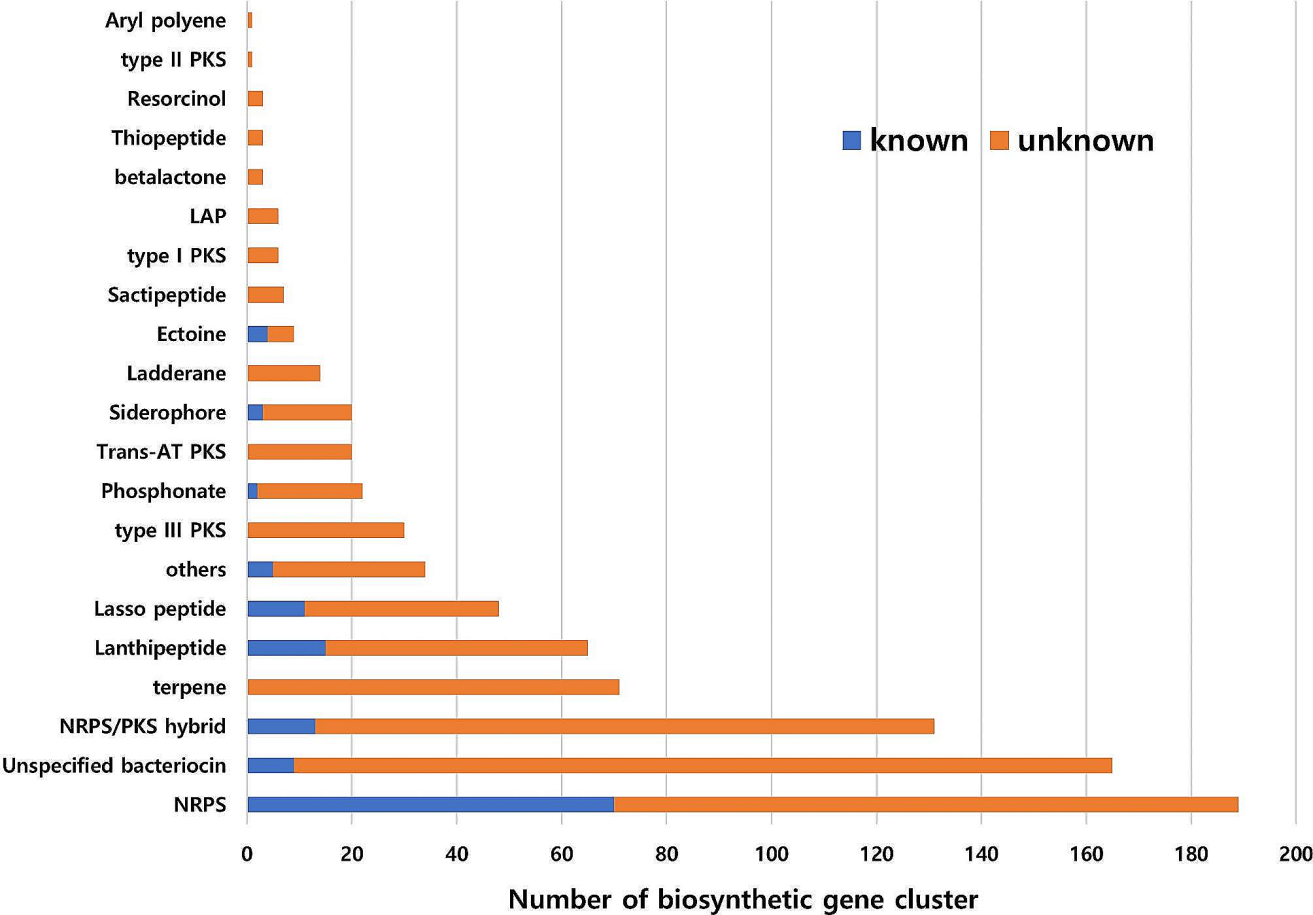
Analysis of biosynthetic gene clusters in 89 genome sequences of *Paenibacillus* strains. The x-axis includes cyclic-lactone-autoinducer, proteusin, redox-cofactor, ranthipeptide, and oligosaccharide. LAP, linear azol(in)e-containing peptide; NRPS, non-ribosomal peptide synthetase; PKS, polyketide synthase

In the order Bacillales, the exploration of BGCs within the family Paenibacillaceae has been notably limited compared to the Bacillaceae family. To address this, we delved into BGCs within *Paenibacillus* species, representative of the Paenibacillaceae family. We obtained 554 *Paenibacillus* genomes from NCBI (February 4, 2020) and classified them into four genome levels based on sequencing data quality and assembly level: complete, chromosome, scaffold, and contig levels, accounting for 81, 8, 197, and 268 sequences, respectively. Further analysis was conducted on 89 genomes at the complete and chromosome levels (Supplementary Table S4). Using antiSMASH, we analyzed the 89 genomes and detected a total of 848 BGCs, with an average of 9.5 BGCs per strain (Fig. 1). Of the 848 BGCs, 716 (84.4%) were classified as unknown and 671 (79.1%) were associated with the production of non-ribosomal peptide synthetases (NRPS), polyketide synthases (PKS), and bacteriocins. Of the 554 *Paenibacillus* strains whose genomes have been deposited at NCBI, we selected 26 strains from culture collections for more in-depth investigation (Table 1).

**Table 1:** Selected *Paenibacillus* strains available in culture collections for an in-depth evaluation

Paenibacillus strains	source	Genome assembly_level	Accession number
*P. alvei* DSM 29	KCTC 3623	Contig	GCF_000293805.1
*P. assamensis* DSM 18,201	KCTC 13,627	Scaffold	GCF_000422445.1
*P. azotifigens* LMG 29,963	KACC 18,967	Scaffold	GCF_008635805.1
*P. borealis* DSM 13,188	KCTC 3805	Complete Genome	GCF_000758665.1
*P. brasilensis* KACC 13,842	KACC 13,842	Complete Genome	GCF_009363115.1
*P. cellulositrophicus* KACC 16,577	KCTC 13,135	Complete Genome	GCF_009363095.1
*P. curdlanolyticus* YK9	KCTC 3759	Scaffold	GCF_000179615.1
*P. daejeonensis* DSM 15,491	KCTC 3745	Scaffold	GCF_000378385.1
*P. donghaensis* KCTC 13,049	KCTC 13,049	Complete Genome	GCF_002192415.1
*P. durus* ATCC 35,681	KCTC 3740	Contig	GCF_000520635.1
*P. ehimensis* NBRC 15,659	KCTC 43,209	Contig	GCF_004000785.1
*P. fonticola* DSM 21,315	KCTC 13,624	Scaffold	GCF_000381905.1
*P. glacialis* DSM 22,343	KCTC 13,874	Contig	GCF_001637205.1
*P. graminis* DSM 15,220	KCTC 13,926	Complete Genome	GCF_000758705.1
*P. harenae* DSM 16,969	KCTC 3951	Scaffold	GCF_000422465.1
*P. jilunlii* DSM 23,019	KACC 16,679	Contig	GCF_001546055.1
*P. kribbensis* AM49	KACC 17,402	Complete Genome	GCF_002240415.1
*P. nuruki* TI45-13ar	KACC 18,728	Contig	GCF_001721045.1
*P. pabuli* NBRC 13,638	KCTC 3398	Contig	GCF_001514495.1
*P. pini* JCM 16,418	KCTC 13,694	Contig	GCF_000576305.1
*P. pinihumi* DSM 23,905	KCTC 13,695	Scaffold	GCF_000422505.1
*P. polymyxa* ATCC 842	KCTC 3627	Scaffold	GCF_000217775.1
*P. stellifer* DSM 14,472	KCTC 3931	Complete Genome	GCF_000758685.1
*P. taiwanensis* DSM 18,679	KCTC 13,628	Scaffold	GCF_000425125.1
*P. thiaminolyticus* NRRL B-4156	KCTC 3764	Contig	GCF_002161855.1
*P. tianmuensis* CGMCC 1.8946	KACC 16,677	Scaffold	GCF_900100345.1

### BGC analysis and antimicrobial activity evaluation of selected *Paenibacillus* strains

The genomic analyses of these 26 selected strains using antiSMASH revealed 312 BGCs, of which 255 consisted of NRPSs, PKSs, and potentially antibacterial bacteriocins (Table 2). Of the 255 BGCs, 221 (86.7%) were identified as unknown. The remaining 57 BGCs comprised ladderanes, siderophores, terpenes, resorcinol, beta-lactones, phosphonates, oligosaccharides, and ectoine. Strains containing five or more unknown BGCs were *P. brasilensis* KACC 13,842, *Paenibacillus ehimensis* NBRC 15,659, *Paenibacillus glacialis* DSM 22,343, *Paenibacillus pinihumi* DSM 23,905, *Paenibacillus polymyxa* ATCC 842, *Paenibacillus thiaminolyticus* NRRL B-4156, and *Paenibacillus tianmuensis* CGMCC 1.8946. Next, the antimicrobial activities of the 26 *Paenibacillus* strains were evaluated against gram-positive *M. luteus* and gram-negative *E. coli* (Table 2). Of the 26 strains, 20 showed antimicrobial activity against *M. luteus*, whereas only six showed antimicrobial activity against *E. coli*. *Paenibacillus assamensis*, *P. brasilensis*, *P. ehimensis*, *Paenibacillus nuruki*, and *P. tianmuensis* exhibited strong antimicrobial activity against *M. luteus*. *P. ehimiensis* and *P. tianmuensis* showed strong antimicrobial activity against both *M. luteus* and *E. coli*. Six strains, including *Paenibacillus alvei*, *Paenibacillus donghaensis*, *Paenibacillus fonticola*, *Paenibacillus harenae*, *Paenibacillus pinihumi*, and *Paenibacillus thiaminolyticus*, showed no antimicrobial activity, suggesting that their BGCs may be silent under standard laboratory conditions as previously reported [21] or the antibiotics produced may be outside the scope of the antibacterial assay in this study.

**Table 2:** Biosynthetic gene cluster (BGC) analysis and antimicrobial activity of selected *Paenibacillus* strains

Paenibacillus strain	Number of BGC^a^			Antimicrobial activity^b^	
	Known	Unknown	Others	Micrococcus luteus	Escherichia coli
*P. alvei* DSM 29	2	16	1	-	-
*P. assamensis* DSM 18,201	0	8	2	+++	+
*P. azotifigens* LMG 29,963	2	10	0	+	-
*P. borealis* DSM 13,188	1	5	1	+	-
*P. brasilensis* KACC 13,842	2	10	2	+++	-
*P. cellulositrophicus* KACC 16,577	0	6	3	+	-
*P. curdlanolyticus* YK9	0	6	4	+	-
*P. daejeonensis* DSM 15,491	0	8	6	+	-
*P. donghaensis* KCTC 13,049	0	10	2	-	-
*P. durus* ATCC 35,681	0	5	2	+	-
*P. ehimensis* NBRC 15,659	2	13	2	+++	+++
*P. fonticola* DSM 21,315	0	8	1	-	-
*P. glacialis* DSM 22,343	0	8	3	+	-
*P. graminis* DSM 15,220	1	10	0	+	+
*P. harenae* DSM 16,969	2	2	4	-	-
*P. jilunlii* KACC 16,679	1	5	0	++	-
*P. kribbensis* AM49	2	9	5	+	-
*P. nuruki* TI45-13ar	1	5	1	+++	-
*P. pabuli* NBRC 13,638	1	7	1	+	-
*P. pini* JCM 16,418	1	6	3	+	-
*P. pinihumi* DSM 23,905	1	19	6	-	-
*P. polymyxa* ATCC 842	5	14	2	++	++
*P. stellifer* DSM 14,472	0	4	1	++	-
*P. taiwanensis* DSM 18,679	1	9	1	+	+
*P. thiaminolyticus* NRRL B-4156	3	12	2	-	-
*P. tianmuensis* CGMCC 1.8946	6	6	2	+++	+++
Total	34	221	57		

^a^BGC analysis was performed using antiSMASH. The known and unknown BGCs are associated with non-ribosomal peptide synthetase, polyketide synthase, and bacteriocin production. Others include ladderane, siderophore, terpene, resorcinol, betalactone, phosphonate, oligosaccharide, and ectoine

^**b**^-. no inhibition; +, < 1 mm zone; ++, 1–3 mm zone; +++ > 3 mm zone of growth inhibition

Of the 26 strains selected, the genome sequences of seven were determined completely. Of the seven strains, *P. brasilensis* was selected for further analysis to discover novel antibiotics derived from *Paenibacillus* species because of its strong antimicrobial activity against *M. luteus*.

### Characterization of a new antibiotic from *P*. *brasilensis*

**Fig. 2:**
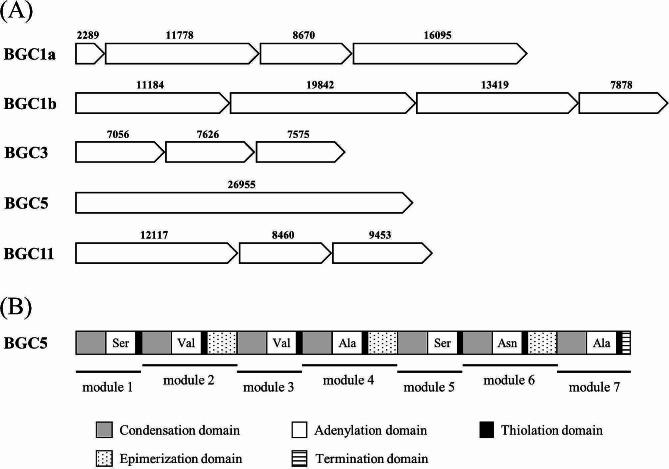
(**A**) BGCs in the genome of *Paenibacillus brasilensis*. The numbers represent the size (in base pairs) of the gene. (**B**) Domain organization of a non-ribosomal peptide synthetase encoded by BGC5. Modules 2, 4, and 6 contain epimerization domains that convert the corresponding amino acid to its D-form. BGC, biosynthetic gene cluster

AntiSMASH analysis revealed the presence of 14 potential BGCs in *P. brasilensis* (Supplementary Fig. S1), of which BGC1, BGC3, BGC5, and BGC11 were classified as new BGCs based on gene structure and annotation. In addition, BGC1 was subclassified into BGC1a and BGC1b because it appeared to comprise two BGCs. Consequently, it was suggested that *P. brasilensis* contained five new BGCs (Fig. 2, A). Among them, BGC5 was annotated as a fusaricidin B-encoding BGC using antiSMASH. However, BGC5-encoded peptide antibiotic (BGC5 antibiotic) consisted of seven amino acids, while fusaricidin B consisted of six amino acids. The amino acid composition of the BGC5 antibiotic was Ser-(D)Val-Val-(D)Ser-Ser-(D)Asn-Ala (Fig. 2, B), whereas that of fusaricidin B was Thr-(D)Val-Val-(D-allo)Thr-(D)Gln-(D)Ala [20]. Therefore, the BGC5 antibiotic was considered a novel antibiotic because its size and amino acid composition are different from those of fusaricidin B. Additionally, AntiSMASH annotated BGC11 as a BGC encoding fusaricidin B (Supplementary Figure S1). BGC11 comprises two separate BGCs: the first is similar to the fusaricidin BGC, and the second is a novel BGC. Upon comparing the first BGC with the fusaricidin BGC from *P. polymyxa* E681, it was found that the first BGC is considered a pseudogene, with 55% of the fusaricidin gene deleted. Consequently, the first BGC was excluded from the antibiotic BGC analysis.

**Fig. 3:**
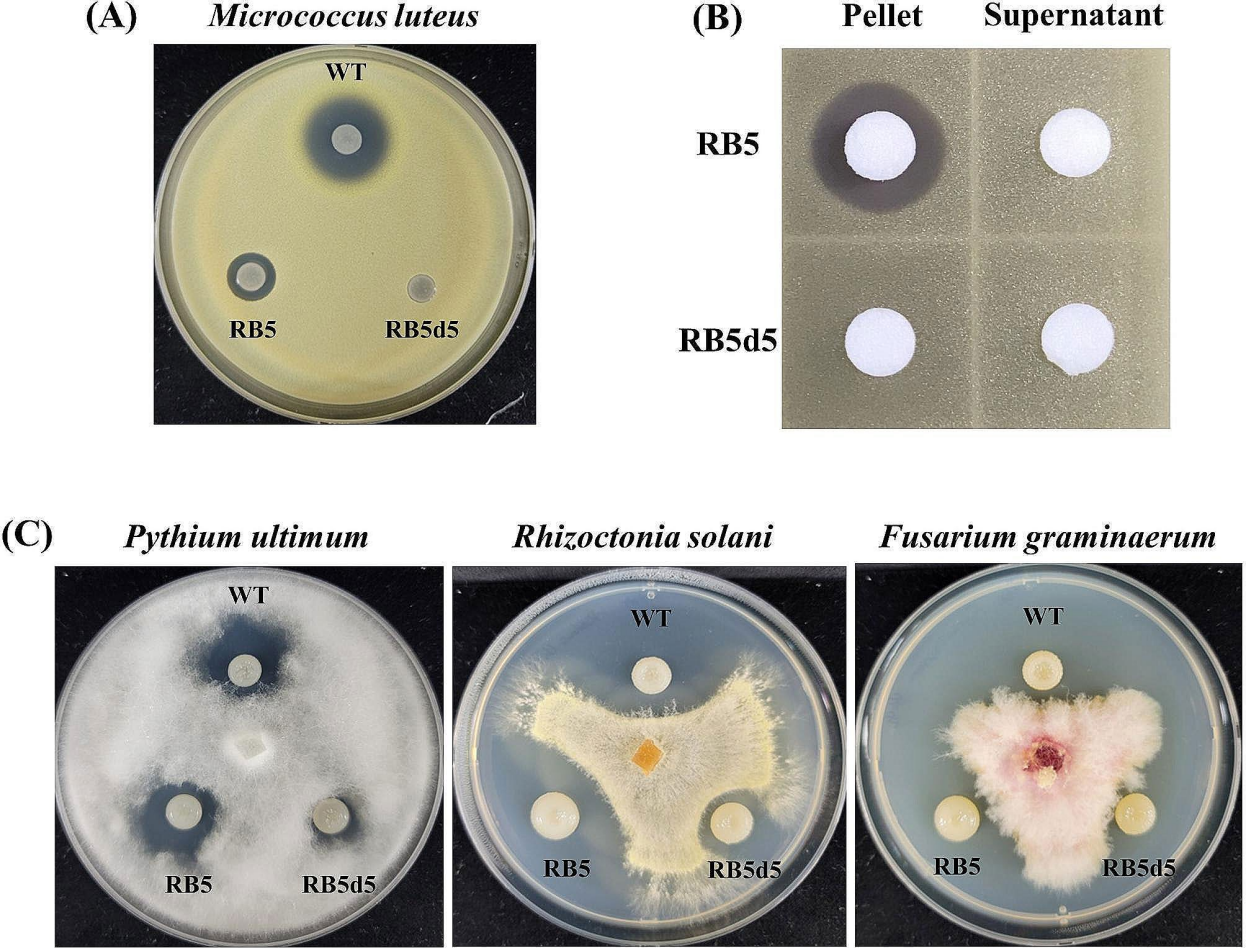
(**A**) Antimicrobial activities of *Paenibacillus brasilensis* and its mutants against *Micrococcus luteus*. (**B**) Antimicrobial activities of the methanol-extracted cell pellets and culture supernatants of RB5 and RB5d5 against *M. luteus*. (**C**) Antifungal activities of *P. brasilensis* and its mutants against fungi. Strain RB5 contains four inactivated BGCs, leaving only BGC5 functional. In RB5d5, all five BGCs are inactivated. BGC, biosynthetic gene cluster

To ensure accurate characterization, the BGC5 antibiotic required dereplication, preventing the influence of other antibiotics. We accomplished this by constructing the *P. brasilensis* RB5 strain, in which four BGCs were disabled to leave only BGC5 active, utilizing a CBE-mediated antibiotic dereplication method [17]. Subsequently, the remaining BGC5 in RB5 was deleted using the BIPS system [18] to construct the RB5d5 strain in which all five BGCs were inactivated. The BIPS system minimizes the potential for secondary mutations on the genome, thus safeguarding genetic integrity through traditional double-crossover recombination. We assessed the antimicrobial activities of wild-type, RB5, and RB5d5 *P. brasilensis* against gram-positive bacteria, gram-negative bacteria, and fungi. The wild-type strain demonstrated antimicrobial activity against gram-positive bacterium *M. luteus* (Fig. 3A) and fungi *P. ultimum*, *R. solani*, and *F. graminearum* (Fig. 3C). However, it proved ineffective against the gram-positive bacterium *B. cereus* and gram-negative strains *E. coli*, *A. baumannii*, and *P. aeruginosa*. The RB5 strain exhibited diminished antibacterial activity against *M. luteus*, while the RB5d5 strain displayed no antimicrobial activity. These findings emphasize the role of the BGC5 antibiotic in combating *M. luteus* and suggest that other antibiotics may contribute to its activity. As for antifungal activity, the RB5 strain showed performance similar to the wild-type strain, whereas the RB5d5 strain demonstrated reduced efficacy. Consequently, the antifungal activity of *P. brasilensis* was mainly dependent on the BGC5 antibiotic, although residual antifungal activity in RB5d5 suggests the presence of other metabolites with similar properties. In summary, BCG5 demonstrated antimicrobial activity against gram positive bacteria and fungi and shared similarities with fusaricidin antibiotics.

**Fig. 4:**
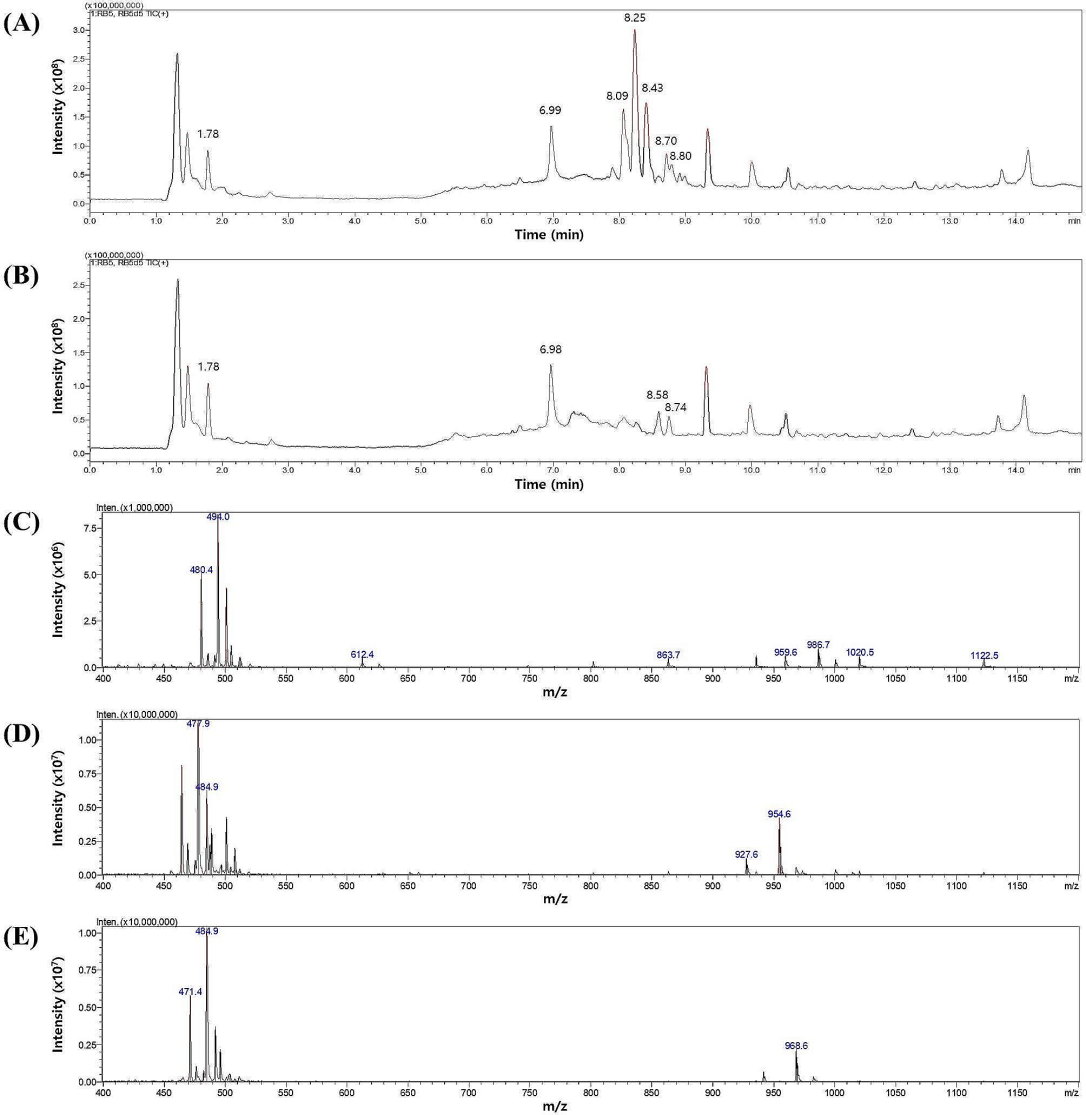
Liquid chromatography analysis of cell pellet methanol extracts of RB5 (**A**) and RB5d5 (**B**) with mass spectrometry data at retention times of 8.09 (**C**), 8.25 (**D**), and 8.43 min (**E**), respectively

For the further characterization of the BGC5 antibiotics, cell pellets and culture supernatants of the RB5 were extracted with methanol and the antimicrobial activities of the extracts were compared to those of the RB5d5 strain. Antimicrobial activity was demonstrated only in the extract from RB5 (Fig. 3B), indicating that BGC5 encodes an antibiotic active against *M. luteus*. LC/MS analysis supported this result, in that the peak corresponding to the BGC5 antibiotic disappeared in the RB5d5 extract (Fig. 4). Interestingly, antimicrobial activity was observed in the extract from the cell pellet of RB5, indicating that the BGC5 antibiotic was bound to the cell wall, similar to fusaricidin antibiotics [20]. Although the size and amino acid composition of the BGC5 antibiotic were different to that of fusaricidin B, both antibiotics shared similar properties in terms of cellular location and antimicrobial spectrum.

**Fig. 5:**
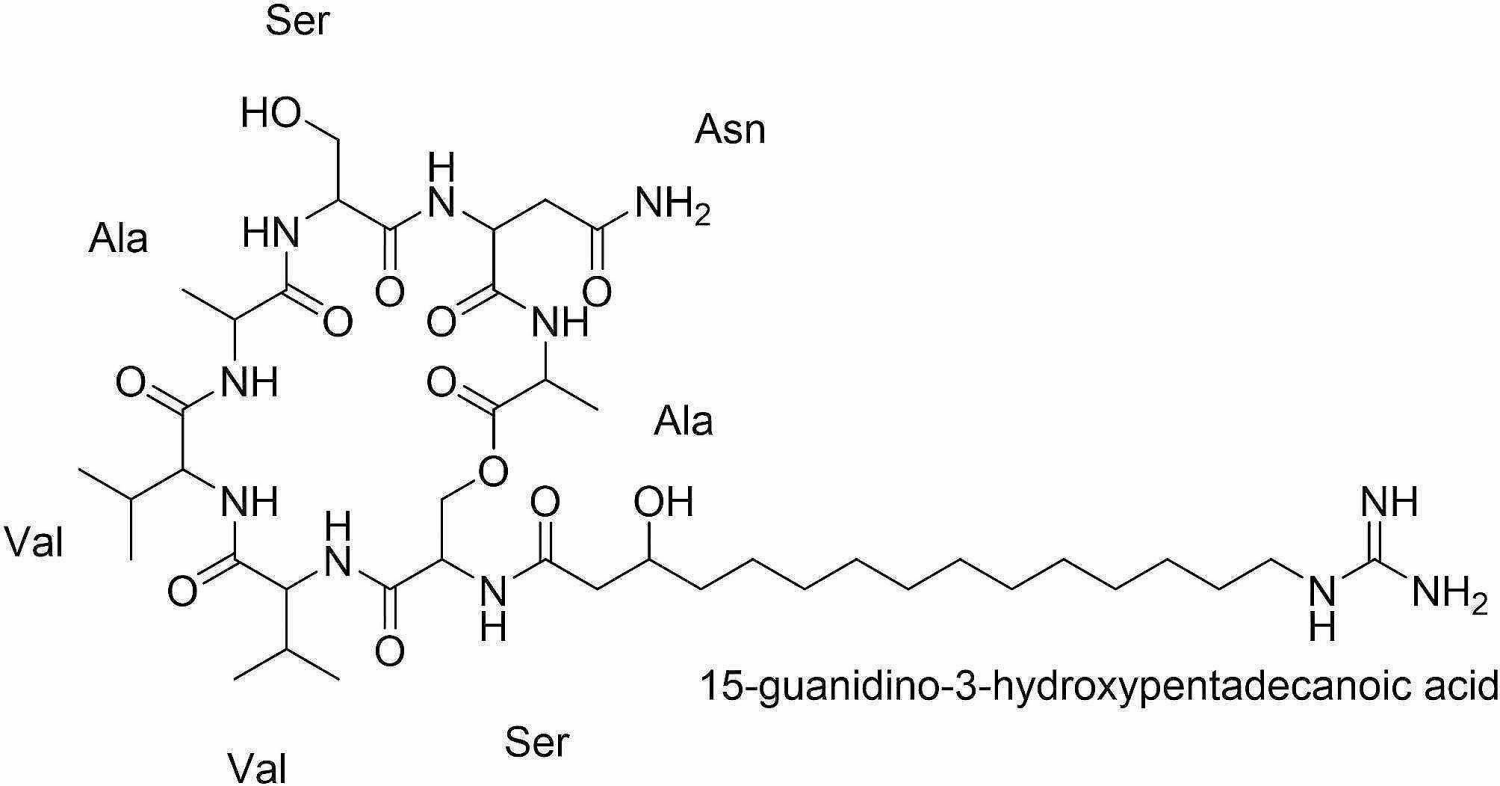
Chemical structure of the BGC5 antibiotic (bracidin) identified using nuclear magnetic resonance spectroscopy

We determined the chemical structure of the BGC5 antibiotic using NMR spectroscopy (Supplementary Fig. S2 and Fig. S3). The structure was found to be a novel cyclic lipopeptide made up of seven amino acids (Fig. 5). The amino acid composition of the BGC5 antibiotic was Ser-(D)Val-Val-(D)Ala-Ser-(D)Asn-Ala, which differed by one amino acid from the predicted composition from the antiSMASH analysis: Ser-(D)Val-Val-(D)Ser-Ser-(D)Asn-Ala. The novel compound, with a molecular weight of 926, was named bracidin.

## Discussion

In classical methods, new antibiotics from natural resources can be identified using multiple processes, including the isolation of pure compounds and subsequent structural analysis. The processes are labor-intensive and time-consuming and are mostly unsuccessful due to the large number of known compounds. As sequencing costs have recently decreased dramatically, genome sequence data is accumulating exponentially [22]. Advanced technologies now facilitate the culture of previously unculturable bacteria, which greatly expands the pool of bacterial genomes [23]. *In silico* genomic analysis tools can mine BGCs quickly and in large quantities, making them a powerful engine in the development of new antibiotics. The most attractive advantage of the tools is that they can predict the novelty of antibiotics without laborious chemical purification and characterization processes [16]. Several BGC databases, such as antiSMASH, ClusterMine360 [24], and IMG-ABC [25] are available to users. Therefore, genome-based antibiotic development strategies can be a useful weapon to combat the threat of AMR by the continuous supply of new antibiotics.

Antibiotics from *Paenibacillus* species, especially *P. polymyxa* E681, which has strong antibacterial and antifungal activities, have been well-studied. E681 produces at least six antibiotics, including polymyxin, fusaricidin, tridecaptin, paenilipoheptin, paenilan, and bacillaene-like antibiotics [26]. Among these, polymyxin is used as a last-resort antibiotic for treating infections caused by multidrug-resistant gram-negative pathogenic bacteria [27]. However, other *Paenibacillus* species remain relatively unexplored. Previous research has explored 36 *Paenibacillus* genomes has identified 188 antimicrobial-encoding gene clusters [13]. Another study analyzed 479 *Paenibacillus* genomes for lanthipeptide mining [28]. In the present study, we conducted a more comprehensive analysis by examining 89 *Paenibacillus* genomes deposited at NCBI. The antiSMASH analysis of the selected 89 genomes identified 848 BGCs, the majority of which (716; 84.4%) were uncharacterized. When the 26 further selected *Paenibacillus* genomes were analyzed using antiSMASH, 221 (86.7%) of 255 BGCs encoding NRPSs, PKSs, and bacteriocins were classified as unknown. The results showed that most BGCs derived from *Paenibacillus* species are uncharacterized, indicating that *Paenibacillus* species represent valuable resources for the discovery of novel antibiotics.

One of the most formidable challenges in antibiotic discovery is dealing with a vast collection of known compounds [2], necessitating the process of antibiotic dereplication to distinguish new compounds. In addition, the genome-based discovery of novel antibiotics requires dereplication because most bacteria contain multiple BGCs and likely produce antibiotic mixtures. Our genome analysis indicated that *Paenibacillus* strains typically contained an average of 9.5 BGCs per strain. Although traditional dereplication methods have improved with advanced analytical techniques [7]; they still require purification steps to obtain pure fractions, making them unsuitable as initial screening methods. Recently, an efficient genetic dereplication method was developed using the CBE system, which can construct a single antibiotic-producing strain by simultaneously inactivating multiple BGCs [17]. However, CBE-based genetic dereplication must overcome the transformation barrier of the wild-type strain because it is difficult to transform foreign DNA into wild-type strains. Recently, it was reported that the broad-host-range conjugation system, MICE, facilitates the efficient use of genome-editing tools in various wild-type *Bacillus* strains [15]. Here, we applied the CBE system to *P. brasilensis* using the MICE system, simultaneously inactivated the four BGCs to construct a single BGC5 antibiotic-producing strain and investigated the antibacterial activity of this new antibiotic. Therefore, we demonstrated that genetic dereplication via the MICE and CBE systems is also possible in *Paenibacillus* species, which can facilitate subsequent processes such as activity-based screening, purification, and characterization of antibiotics.

Despite the wealth of information unlocked by large-scale genomic analyses, many BGCs remain inactive or silent under standard laboratory conditions [21]. As our results showed, six *Paenibacillus* strains, including *P. alvei*, *P. donghaensis*, *P. fonticola*, *P. harenae*, *P. pinihumi*, and *P. thiaminolyticus*, possessed one or more BGCs; however, they did not show antibacterial activity against *M. luteus* or *E. coli*. It is possible that BGCs in these six strains as well as in many other *Paenibacillus* strains are cryptic. Therefore, the activation of silent BGCs is important for the discovery of new antibiotics. Several methods have been developed to activate silent BGCs, including the heterologous expression of BGCs in surrogate hosts, homologous expression under different fermentation conditions, screening of elicitors, co-cultivation, ribosome and RNA polymerase engineering, regulatory gene activation, histone modification, and metabolic remodeling for precursor supply [29]. The heterologous expression of BGCs in surrogate hosts is limited by difficulties faced in cloning BGCs owing to their large size, incompatible regulatory systems, and absence of biosynthetic precursors or essential enzymes. Homologous expression, inducer screening, and co-culture methods can facilitate the production of antibiotic mixtures, suggesting that antibiotic dereplication is still required to characterize specific compounds. Ribosome and RNA polymerase engineering, regulatory gene activation, histone modification, and metabolic remodeling methods require both antibiotic dereplication and genome editing in wild-type strains. In this study, we constructed a single antibiotic-producing strain using MICE- and CBE-mediated genetic dereplication of antibiotics, which facilitated the characterization of a new antibiotic. Therefore, the construction of a single antibiotic-producing strain, as performed in this study, prior to the activation of silent BGCs, will greatly accelerate the genome-based development of new antibiotics.

## Conclusions

Our analysis of 89 *Paenibacillus* genomes revealed 848 BGCs, with a significant proportion being uncharacterized. This highlights the potential of *Paenibacillus* species for genome-based antibiotic discovery. Moreover, we successfully constructed a single antibiotic-producing *P. brasilensis* strain using the MICE and CBE systems, which greatly expedited the characterization of the new antibiotic. This approach is not exclusive to *Paenibacillus* and can be extended to other genera within the Bacillaceae and Paenibacillaceae families. This strategy represents a promising avenue for antibiotic discovery, including the identification of new BGCs via genome analysis, construction of single antibiotic-producing strains using MICE and CBE systems, and subsequent characterization of novel antibiotics; it can be applied to unexplored strains of other genera belonging to the families Bacillaceae and Paenibacillaceae as well as other members of the order Bacillales.

### Electronic supplementary material

Below is the link to the electronic supplementary material.

Supplementary Material 1

## Data Availability

The datasets generated and/or analysed during the current study are available in the GenBank repository. The accession numbers for the Paenibacillus genomes are provided in the Supplementary Information.

## Abbreviations

BGC: Biosynthetic gene cluster
CBE: Cytosine base editor
ESIMS: Electrospray ionization mass spectrometry
NMR: Nuclear magnetic resonance
AMR: Antimicrobial resistance
NCBI: National Center for Biotechnology Information
KACC: Korean Agricultural Culture Collection
KCTC: Korean Collection for Type Cultures
LB: Luria-Bertani
TSB: Tryptic soy broth
TSA: Tryptic soy agar
ATCC: American Type Culture Collection
PDA: Potato dextrose agar
sgRNA: Single-guide RNA
MICE: Modified integrative and conjugative element
BIPS: *Bacillus* integrative plasmid combining a synthetic gene circuit
*cat*: Chloramphenicol resistance gene
LC/MS: Liquid chromatography–mass spectrometry
UPLC BEH: Ultra performance liquid chromatography ethylene-bridged hybrid
HPLC: High-performance liquid chromatography
DMSO: Dimethyl sulfoxide
NRPS: Non-ribosomal peptide synthetases
PKS: Polyketide synthases
