# Supplementary material for: Mining biosynthetic gene clusters in Paenibacillus genomes to discover novel antibiotics
Source: BMC Microbiol. 2024 Jun 27;24:226. doi: 10.1186/s12866-024-03375-5 (PMC11210098; doi:10.1186/s12866-024-03375-5)
Supplement: Supplementary file 1 — Supplementary Material 1 [file 12866_2024_3375_MOESM1_ESM.docx]

**Supplementary information**

**Mining biosynthetic gene clusters in *Paenibacillus* genomes to discover novel antibiotics**

**Man Su Kim^1,2†^, Da-Eun Jeong^1†^, Jun-Pil Jang^3†^, Jae-Hyuk Jang^3,4*^, and Soo-Keun Choi^1,2*^**

^1^Infectious Disease Research Center, Korea Research Institute of Bioscience and Biotechnology (KRIBB), Daejeon, Republic of Korea,

^2^Department of Biosystems and Bioengineering, KRIBB School of Biotechnology, University of Science and Technology (UST), Daejeon, Republic of Korea,

^3^Chemical Biology Research Center, Korea Research Institute of Bioscience and Biotechnology, Cheongju, Republic of Korea

^4^Department of Applied Biological Engineering, KRIBB School of Biotechnology, University of Science and Technology (UST), Daejeon, Republic of Korea

The following Supplementary information is available for this article:

**Table S1.** Bacterial strains used in this study**.**

**Table S2.** Plasmids used in this study.

**Table S3.** Oligonucleotides and primers used in this study.

**Table S4.** *Paenibacillus* genomes selected for biosynthetic gene cluster analysis.

**Fig. S1.** AntiSMASH analysis of *Paenibacillus brazilensis* genome to identify gene clusters for secondary metabolites.

**Fig. S2.** 1D NMR spectrum of the novel compound.

**Fig. S3.** 2D NMR spectrum of the novel compound.

**References**

**Supplementary Table S1.** Bacterial strains used in this study.

| **Strain** | **Genotype** | **Reference** |
| --- | --- | --- |
| ***Escherichia coli*** |  |  |
| MC1061 | *araD*139 △(*araA*-*leu*)7697 △(*lac*)X74 *galK*16 *galE*15(*GalS*) lambda- e14- *mcrA0* *relA1* *rpsL*150(strR) *spoT1* *mcrB1* hsdR2 | Laboratory stock |
| ***Bacillus subtilis*** |  |  |
| BS5918 | *B. subtilis* 168 △*rapI*-*attR*::*hyg*^R^ △*alrA*::Sp^R^-P*_xylA_*-*rapI* △*oriT*_ICE_ △*sigK* | [1] |
| MICEaRep | BS5918 △*amyE*::P*_ara_*-*rep* | [2] |
| ***Paenibacillus*** |  |  |
| *P. alvei* DSM 29 |  | KCTC 3623 |
| *P. assamensis* DSM 18201 |  | KCTC 13627 |
| *P. azotifigens* LMG 29963 |  | KACC 18967 |
| *P. borealis* DSM 13188 |  | KCTC 3805 |
| *P. brasilensis* KACC 13842 |  | KACC 13842 |
| *P. cellulositrophicus* KACC 16577 |  | KCTC 13135 |
| *P. curdlanolyticus* YK9 |  | KCTC 3759 |
| *P. daejeonensis* DSM 15491 |  | KCTC 3745 |
| *P. donghaensis* KCTC 13049 |  | KCTC 13049 |
| *P. durus* ATCC 35681 |  | KCTC 3740 |
| *P. ehimensis* NBRC 15659 |  | KCTC 43209 |
| *P. fonticola* DSM 21315 |  | KCTC 13624 |
| *P. glacialis* DSM 22343 |  | KCTC 13874 |
| *P. graminis* DSM 15220 |  | KCTC 13926 |
| *P. harenae* DSM 16969 |  | KCTC 3951 |
| *P. jilunlii* KACC 16679 |  | KACC 16679 |
| *P. kribbensis* AM49 |  | KACC 17402 |
| *P. nuruki* TI45-13ar |  | KACC 18728 |
| *P. pabuli* NBRC 13638 |  | KCTC 3398 |
| *P. pini* JCM 16418 |  | KCTC 13694 |
| *P. pinihumi* DSM 23905 |  | KCTC 13695 |
| *P. polymyxa* ATCC 842 |  | KCTC 3627 |
| *P. stellifer* DSM 14472 |  | KCTC 3931 |
| *P. taiwanensis* DSM 18679 |  | KCTC 13628 |
| *P. thiaminolyticus* NRRL B-4156 |  | KCTC 3764 |
| *P. tianmuensis* CGMCC 1.8946 |  | KACC 16677 |
| RB5 | *P. brasilensis* △BGC1a △BGC1b △BGC3 △BGC11 | This study |
| RB5d5 | RB5 △BGC5::Cm | This study |
| **Indicator for antimicrobial activity assay** |  |  |
| *Acinetobacter baumannii* |  | ATCC 19606 |
| *E. coli* |  | KCTC 22003 |
| *Pseudomonas aeruginosa* |  | ATCC 27853 |
| *Bacillus cereus* |  | ATCC 4342 |
| *Micrococcus luteus* |  | KCTC 2177 |
| *Pythium ultimum* |  | KACC 40705 |
| *Fusarium graminearum* |  | KACC 41040 |
| *Rhizoctonia solani* |  | KACC 40146 |

**Supplementary Table S2.** Plasmids used in this study.

| **Plasmid** | **Description** | **Reference** |
| --- | --- | --- |
| pMGoldi-sCBE4 | pMGold-sCBE4 derivative containing *oriT*_ICE_ (for MICE) instead of *oriT* (for EBC) | [2] |
| pMisCBE4-RB5 | pMGoldi-*sCBE4* with sgRNA: BGC1a, BGC1b, BGC3, and BGC11 | This study |
| pSGC4iN | pSGC2iN derivative containing synsthtic gene circuit | [3] |
| pSGC4iN-RB5d | pSGC4iN derivative, inserting P*_spac_*-*cat* at BGC5 locus of *P. brasilensis* | This study |

**Supplementary Table S3.** Oligonucleotides and primers used in this study

| **Oligonucleotide** | **Sequence (5’ to 3’)** | **Purpose** |
| --- | --- | --- |
| 1a-BraPK-gRF | ATTG**TTCAAGCCGGAGCAGATCAG** | Oligonucleotides for cloning of synthetic gRNA sequence used in this study |
| 1a-BraPK-gRR | AAAC**CTGATCTGCTCCGGCTTGAA** |  |
| 1b-BraPK-gRF | ATTG**TCAGAGTCCCCGACTTCTTA** |  |
| 1b-BraPK-gRR | AAAC**TAAGAAGTCGGGGACTCTGA** |  |
| 3-BraNRP-gRF | ATTG**CAGCGAGCTTTTGAGATACT** |  |
| 3-BraNRP-gRR | AAAC**AGTATCTCAAAAGCTCGCTG** |  |
| 11-BraNRP-gRF | ATTG**GCAGGCAGGAAGCGAATACG** |  |
| 11-BraNRP-gRR | AAAC**CGTATTCGCTTCCTGCCTGC** |  |
| **Primer** | **Sequence (5’ to 3’)** | **Purpose** |
| BB-vec-sgF | GGAAGATCTGGTCTCCATTGAGCCAGCAAGACAGCGATAA | Primers for multiplex gRNA cloning |
| Bsa-sgR1 | ACGGTCTCCCCTTAAAAAAAGCACCGACTCGGTG |  |
| Bsa-sgF1 | CTGGTCTCCAAGGAGCCAGCAAGACAGCGATAA |  |
| Bsa-sgR2 | ACGGTCTCCGAGTAAAAAAAGCACCGACTCGGTG |  |
| Bsa-sgF2 | CTGGTCTCCACTCAGCCAGCAAGACAGCGATAA |  |
| Bsa-sgR3 | ACGGTCTCCTCCTAAAAAAAGCACCGACTCGGTG |  |
| Bsa-sgF3 | CTGGTCTCCAGGAAGCCAGCAAGACAGCGATAA |  |
| SCBB-vec-sgR | GGAGTCGACATCGATGGATCCGGTCTCCAAACAAAAAAAGCACCGACTCGGTG |  |
| 1G-BRB5-FF | CTGGTCTCCATTGTCCTTTGCGAATATCCATGC | Primers for amplifying homologous arm fragments to delete target gene |
| 1G-BRB5-FR | ACGGTCTCCCCTTAGTAGCCAAACCTTTGCCGA |  |
| 3GX-BRB5-BF | CTGGTCTCCACTCATGCCTTAAAGCGCTCCATC |  |
| 3GX-BRB5-BR | ACGGTCTCCAAACCAGATCATTGACTTCATCGC |  |
| Pspac-F | CTGGTCTCCAAGGTACACAGCCCAGTCCAGACT | Primers for amplifying P*_spac_*-*cat* |
| cat-R | ACGGTCTCCGAGTGCGAATGGCGACTAACGGGG |  |
| 1a-BraPK-F | TGCCTTGATCCAAACAGTCA |  |
| 1a-BraPK-R | TCCGGAAAACACTTCATACG |  |
| 1b-BraPK-F | GGCTATTCCGGTCAATTCTC |  |
| 1b-BraPK-R | GCCGCACCTGTATATCTCAA |  |
| 3-BraNRP-F | CTGTATAATGCCCTTCATGATC |  |
| 3-BraNRP-R | GCCTATATTATCTATACCTCAGG |  |
| 11-BraNRP-F | GATATCCGGACGAACATCAG |  |
| 11-BraNRP-R | TCCTAGCGCTCAGACGGATC |  |
| 1a-BraPK-seqF | CCGCACAAATAATCGCCTTG |  |
| 1b-BraPK-seqF | ATCCTTATGTCAACACCAAC |  |
| 3-BraNRP-seqF | CTCCTTCACCGCCTATATAC |  |
| 11-BraNRP-seqF | AACCTAGCGAGCGGATGTAC |  |

Underlined sequences are the restriction enzyme sites.

Bolded sequences represent the 20 bp synthetic gRNA.

**Supplementary Table S4.** *Paenibacillus* genomes selected for biosynthetic gene cluster analysis.

| **Organism name** | **Infraspecific_name** | **Genome assembly_level** | **Accession number** |
| --- | --- | --- | --- |
| *P. alvei* |  | Chromosome | GCF_900519125.1 |
| *P. antarcticus* | KACC 11469 | Complete Genome | GCF_008369725.1 |
| *P. baekrokdamisoli* | KCTC 33723 | Complete Genome | GCF_003945345.1 |
| *P. beijingensis* | DSM 24997 | Complete Genome | GCF_000961095.1 |
| *P. borealis* | DSM 13188 | Complete Genome | GCF_000758665.1 |
| *P. bovis* | BD3526 | Complete Genome | GCF_001421015.2 |
| *P. brasilensis* | KACC 13842 | Complete Genome | GCF_009363115.1 |
| *P. cellulositrophicus* | KACC 16577 | Complete Genome | GCF_009363095.1 |
| *P. chitinolyticus* | KCCM 41400 | Complete Genome | GCF_004117095.1 |
| *P. crassostreae* | LPB0068 | Complete Genome | GCF_001857945.1 |
| *P. donghaensis* | KCTC 13049 | Complete Genome | GCF_002192415.1 |
| *P. durus* | DSM 1735 | Complete Genome | GCF_000756615.1 |
| *P. durus* | ATCC 35681 | Complete Genome | GCF_000993825.1 |
| *P. glucanolyticus* | 5162 | Chromosome | GCF_001632305.1 |
| *P. glucanolyticus* | W10507 | Chromosome | GCF_003033945.1 |
| *P. graminis* | DSM 15220 | Complete Genome | GCF_000758705.1 |
| *P. guangzhouensis* | KCTC 33171 | Complete Genome | GCF_009363075.1 |
| *P. ihbetae* | IHBB 9852 | Complete Genome | GCF_002741055.1 |
| *P. kribbensis* | AM49 | Complete Genome | GCF_002240415.1 |
| *P. larvae* subsp. *larvae* | ATCC 9545 | Complete Genome | GCF_002003265.1 |
| *P. larvae* subsp. *larvae* | ERIC_I | Complete Genome | GCF_002951875.1 |
| *P. larvae* subsp. *larvae* | Eric_III | Complete Genome | GCF_002951915.1 |
| *P. larvae* subsp. *larvae* | Eric_IV | Complete Genome | GCF_002951935.1 |
| *P. larvae* subsp. *larvae* | DSM 25430 | Complete Genome | GCF_000511405.1 |
| *P. larvae* subsp. *larvae* | DSM 25430; ERIC_II | Complete Genome | GCF_002951895.1 |
| *P. larvae* subsp. *pulvifaciens* | ATCC 13537 | Complete Genome | GCF_002007765.1 |
| *P. larvae* subsp. *pulvifaciens* | CCM 38 | Complete Genome | GCF_002043025.1 |
| *P. larvae* subsp. *pulvifaciens* | SAG 10367 | Complete Genome | GCF_002082155.1 |
| *P. lautus* | E7593-69 | Complete Genome | GCF_003590055.1 |
| *P. lentus* | DSM 25539 | Complete Genome | GCF_003931855.1 |
| *P. mucilaginosus* | 3016 | Complete Genome | GCF_000250655.1 |
| *P. mucilaginosus* | K02 | Complete Genome | GCF_000258535.2 |
| *P. mucilaginosus* | KNP414 | Complete Genome | GCF_000218915.1 |
| *P. naphthalenovorans* | 32O-Y | Complete Genome | GCF_001465255.1 |
| *P. odorifer* | DSM 15391 | Complete Genome | 1GCF_000758725.1 |
| *P. odorifer* | CBA7130 | Complete Genome | GCF_003255855.1 |
| *P. odorifer* |  | Complete Genome | GCF_902386945.1 |
| *P. peoriae* | HS311 | Complete Genome | GCF_001272655.2 |
| *P. physcomitrellae* | XB | Chromosome | GCF_002240225.1 |
| *P. polymyxa* | CF05 | Chromosome | GCF_000785455.1 |
| *P. polymyxa* | Sb3-1 | Complete Genome | GCF_000819665.1 |
| *P. polymyxa* | J | Complete Genome | GCF_001719045.1 |
| *P. polymyxa* | YC0136 | Complete Genome | GCF_001874405.2 |
| *P. polymyxa* | YC0573 | Complete Genome | GCF_001874425.3 |
| *P. polymyxa* | ATCC 15970 | Complete Genome | GCF_001922145.1 |
| *P. polymyxa* | HY96-2 | Complete Genome | GCF_002893885.1 |
| *P. polymyxa* | ZF129 | Complete Genome | GCF_006274405.1 |
| *P. polymyxa* | ZF197 | Complete Genome | GCF_007858415.1 |
| *P. polymyxa* | CR1 | Complete Genome | GCF_000507205.3 |
| *P. polymyxa* | E681 | Complete Genome | GCF_000146875.3 |
| *P. polymyxa* | M1 | Complete Genome | GCF_000237325.1 |
| *P. polymyxa* | SC2 | Complete Genome | GCF_000164985.3 |
| *P. polymyxa* | SQR21 | Complete Genome | GCF_000597985.1 |
| *P. psychroresistens* | ML311-T8 | Complete Genome | GCF_009728935.1 |
| *P. riograndensis* | SBR5 | Complete Genome | GCF_000981585.1 |
| *P. sabinae* | T27 | Complete Genome | GCF_000612505.1 |
| *Paenibacillus* sp*.* | 18JY67-1 | Complete Genome | GCF_003952225.1 |
| *Paenibacillus* sp. | 32O-W | Complete Genome | GCF_001465275.1 |
| *Paenibacillus* sp. | 37 | Complete Genome | GCF_008386395.1 |
| *Paenibacillus* sp. | B01 | Complete Genome | GCF_009649995.1 |
| *Paenibacillus* sp. | BIHB4019 | Complete Genome | GCF_002741035.1 |
| *Paenibacillus* sp. | CAA11 | Complete Genome | GCF_003060825.1 |
| *Paenibacillus* sp. | Cedars | Complete Genome | GCF_003184205.1 |
| *Paenibacillus* sp. | DCT19 | Complete Genome | GCF_003268635.1 |
| *Paenibacillus* sp. | FSL H7-0357 | Complete Genome | GCF_000758525.1 |
| *Paenibacillus* sp. | FSL H7-0737 | Complete Genome | GCF_000758545.1 |
| *Paenibacillus* sp. | FSL P4-0081 | Complete Genome | GCF_000758565.1 |
| *Paenibacillus* sp. | FSL R5-0345 | Complete Genome | GCF_000758585.1 |
| *Paenibacillus* sp. | FSL R5-0912 | Complete Genome | GCF_000758605.1 |
| *Paenibacillus* sp. | FSL R7-0273 | Complete Genome | GCF_000758625.1 |
| *Paenibacillus* sp. | FSL R7-0331 | Complete Genome | GCF_000758645.1 |
| *Paenibacillus* sp. | FW100M-2 | Complete Genome | GCF_004135365.1 |
| *Paenibacillus* sp. | HB172198 | Complete Genome | GCF_005577435.1 |
| *Paenibacillus* sp. | IHB B 3084 | Complete Genome | GCF_001447315.1 |
| *Paenibacillus* sp. | 10380 | Complete Genome | GCF_000949425.1 |
| *Paenibacillus* sp. | JDR-2 | Complete Genome | GCF_000023585.1 |
| *Paenibacillus* sp. | lzh-N1 | Complete Genome | GCF_002872435.1 |
| *Paenibacillus* sp. | M-152 | Complete Genome | GCF_003856555.1 |
| *Paenibacillus* sp. | MBLB1234 | Complete Genome | GCF_003991425.1 |
| *Paenibacillus* sp. | RUD330 | Chromosome | GCF_002243345.1 |
| *Paenibacillus* sp. | Y412MC10 | Complete Genome | GCF_000024685.1 |
| *P. stellifer* | DSM 14472 | Complete Genome | GCF_000758685.1 |
| *P. swuensis* | DY6 | Complete Genome | GCF_001644605.1 |
| *P. terrae* | HPL-003 | Complete Genome | GCF_000235585.1 |
| *P. thiaminolyticus* | NRRL B-4156 | Chromosome | GCF_007066085.1 |
| *P. thiaminolyticus* | Mbale | Chromosome | GCF_007066225.1 |
| *P. xylanexedens* | PAMC 22703 | Complete Genome | GCF_001908275.1 |
| *P. xylanilyticus* | W4 | Complete Genome | GCF_009664365.1 |
| *P. yonginensis* | DCY84 | Complete Genome | GCF_001685395.1 |


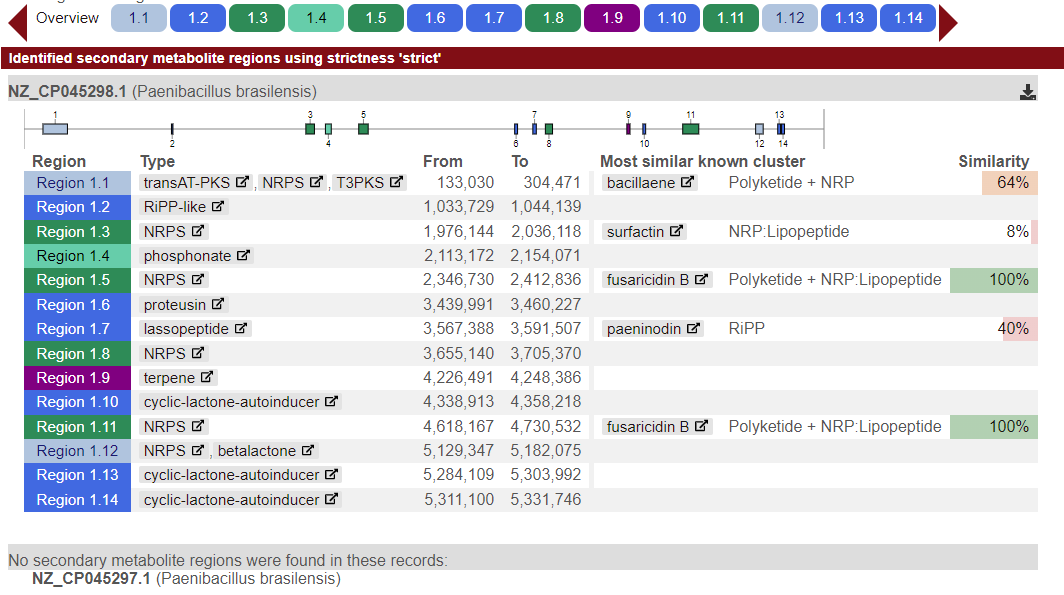


**Fig. S1.** AntiSMASH analysis of *Paenibacillus brazilensis* genome to identify gene clusters for secondary metabolites.

**
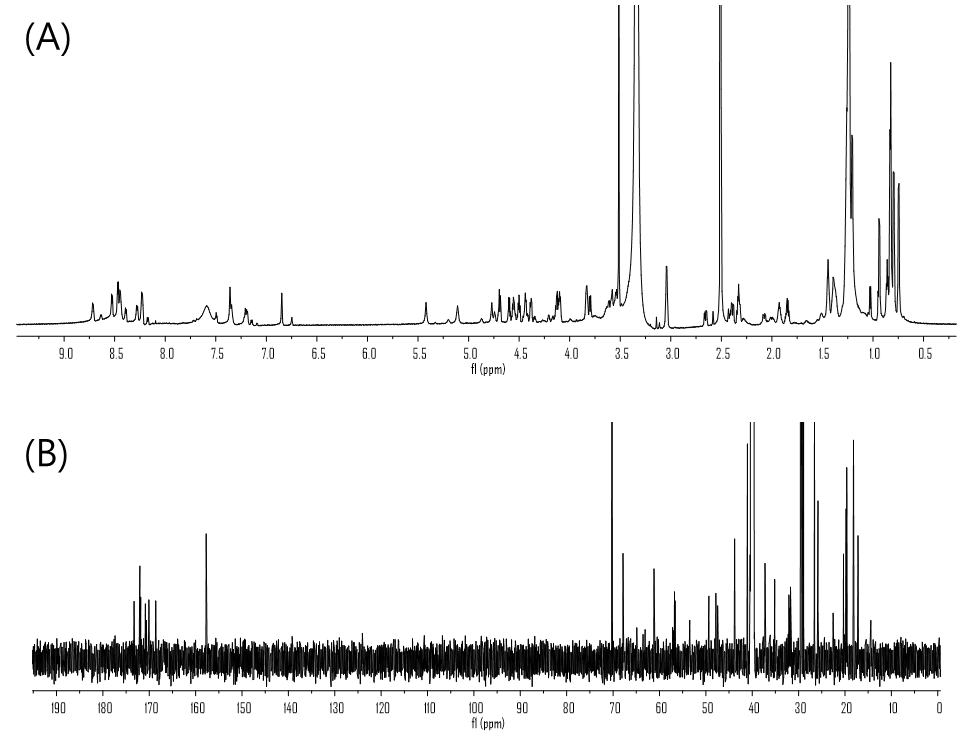
**

**Fig. S2.** 1D NMR spectrum of the novel compound (MW 926). (A) ^1^H NMR spectrum (900 MHz) of the compound in DMSO-*d*_6_. (B) ^13^C NMR spectrum (225 MHz) of the compound in DMSO-*d*_6_.

**
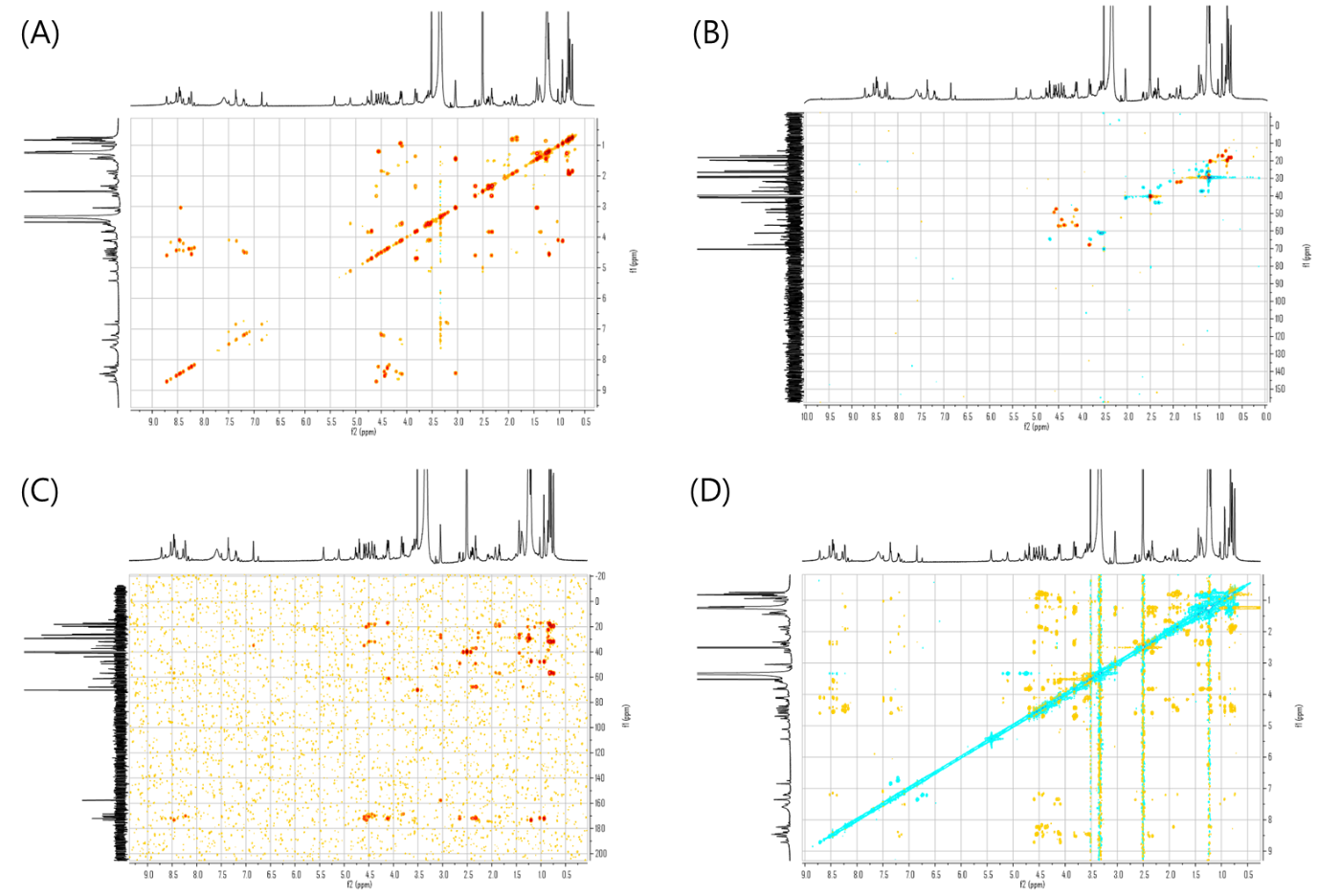
**

**Fig. S3.** 2D NMR spectrum of the novel compound. (A) COSY spectrum of the compound in DMSO-*d*_6_. (B) HSQC-DEPT spectrum of the compound in DMSO-*d*_6_. (C) HMBC spectrum of the compound in DMSO-*d*_6_. (D) ROESY spectrum of the compound in DMSO-*d*_6_.

**References**

1. Jeong DE, Kim MS, Kim HR, Choi SK. Cell Factory Engineering of Undomesticated *Bacillus* Strains Using a Modified Integrative and Conjugative Element for Efficient Plasmid Delivery. Front Microbiol. 2022;13:802040.

2. Kim MS, Kim HR, Jeong DE, Choi SK. Cytosine Base Editor-Mediated Multiplex Genome Editing to Accelerate Discovery of Novel Antibiotics in *Bacillus subtilis* and *Paenibacillus polymyxa*. Front Microbiol. 2021;12:691839.

3. Kim MS, Jeong DE, Choi SK. *Bacillus* integrative plasmid system combining a synthetic gene circuit for efficient genetic modifications of undomesticated *Bacillus* strains. Microb Cell Fact. 2022;21(1):259.
